# A primer set for the rapid isolation of scFv fragments against cell surface antigens from immunised rats

**DOI:** 10.1038/s41598-020-76069-3

**Published:** 2020-11-05

**Authors:** Francesco Nannini, Farhaan Parekh, Patrycja Wawrzyniecka, Leila Mekkaoui, Matteo Righi, Fatemeh V. Dastjerdi, Jenny Yeung, Claire Roddie, Yuchen Bai, Biao Ma, Mathieu Ferrari, Shimobi Onuoha, Kerry Chester, Martin Pule

**Affiliations:** 1 Research Department of Haematology, UCL Cancer Institute, 72 Huntley Street, London, WC1E 6DD UK; 2 Research Department of Oncology, UCL Cancer Institute, London, UK; 3 Autolus Therapeutics, London, UK

**Keywords:** Antibody generation, Membrane proteins, Immunotherapy

## Abstract

Antibody phage display is a powerful platform for discovery of clinically applicable high affinity monoclonal antibodies against a broad range of targets. Libraries generated from immunized animals offer the advantage of in vivo affinity-maturation of V regions prior to library generation. Despite advantages, few studies have described isolation of antibodies from rats using immune phage display. In our study, we describe a novel primer set, covering the full rat heavy chain variable and kappa light chain variable regions repertoire for the generation of an unbiased immune libraries. Since the immune repertoire of rats is poorly understood, we first performed a deep sequencing analysis of the V(D)J regions of VH and VLK genes, demonstrating the high abundance of IGVH2 and IGVH5 families for VH and IGVLK12 and IGVLK22 for VLK. The comparison of gene’s family usage in naïve rats have been used to validate the frequency’s distribution of the primer set, confirming the absence of PCR-based biases. The primers were used to generate and assemble a phage display library from human CD160-vaccinated rats. CD160 represents a valid therapeutic target as it has been shown to be expressed on chronic lymphocytic leukaemia cells and on the surface of newly formed vessels. We utilised a novel phage display panning strategy to isolate a high affinity pool (KD range: 0.399–233 nM) of CD160 targeting monoclonal antibodies. Subsequently, identified binders were tested for function as third generation Chimeric Antigen Receptors (CAR) T cells demonstrating specific cytolytic activity. Our novel primer set coupled with a streamlined strategy for phage display panning enable the rapid isolation and identification of high affinity antibodies from immunised rats. The therapeutic utility of these antibodies was demonstrated in CAR format.

## Introduction

Single-chain variable fragment (scFv) immune phage libraries are convenient tools for antibody discovery. Immune libraries are constructed by incorporating scFv derived from an immunized animal into a phage display system. Panning of an immune scFv phage library combines advantages of both hybridoma and phage display methodologies and can quickly identify multiple diverse binders^1,2^.

Immune libraries are typically derived from immunized mice; but several species have served as the source of variable region diversity for antibody phage libraries including mouse, rabbit, and human^3–5^. However, the generation of rat immune libraries is still relatively unstudied^6,7^, despite rats having several advantages for this application^8,9^. For instance, rats typically undergo rapid seroconversion, have a large set of variable chains and can generate unique binders. Here, we describe a primer set for rat immune library generation which covers all rat germline heavy and kappa light chain variable genes. We exclude bias by comparing deep sequencing of pooled amplified variable regions with rat variable sequence usage.

To determine how well our primers perform, we generated binders against CD160 as a test antigen. CD160 is a GPI-anchored protein with potential to be a useful cancer target^10,11^; it has been detected on endothelial cells of newly formed blood vessels in human colon carcinoma and mouse B16 melanoma but not in vessels of healthy tissues^12,13^ CD160 has been also shown to be aberrantly expressed on B cell malignancies such as B cell chronic lymphocytic leukaemia (B-CLL) and hairy cell leukaemia (HCL)^14,15^. Following successful identification of several anti-CD160 binders, we went on to determine how well the discovered binders function when incorporated into Chimeric Antigen Receptor (CAR) format.

In the present study we have generated a rat immune phage display library for human CD160 through genetic vaccination and v-region amplification with a novel primer set. Antibodies obtained after phage selection on antigen-coated magnetic beads were characterized and studied in functional assays. The identified high affinity antibodies were used to construct 3rd generation CARs able to eradicate CD160 positive cells. This work provides a method for the rapid isolation of specific high affinity antibodies from immunized rats for use in such applications as CAR generation.

## Results

### Design of degenerate primer library for rat VH and VL germline genes

The IMGT database^16^ lists 232 *Rattus norvegicus* germline heavy variable (VH) sequences with 13 families, and 164 kappa light variable (VLK) sequences in 21 families. We designed 39 forward primers which anneal to the VH genes and 29 to the variable kappa light chains. Predicted pseudogenes and not in-frame Open Reading Frames (ORFs) were excluded. In rodents, the kappa light chain is predominantly used, with only 5% of mouse immunoglobulin expressing the lambda light chain, hence primers for the lambda light chain were not designed^17^.

Three strategies allowed the size of the primer set to be kept to a minimum: (1) The similarity shared within each family at the start of the VH genes allowed the design of several primers which cover many functional ORFs. (2) In addition, judicious incorporation of up to 3 ambiguous DNA nucleotides per primer increased the numbers of genes primed per primer; (3) Locked Nucleic Acids (LNA)^18^ were incorporated to anneal to conserved residues of the variable chains’ genes. This avoided the need to design long primers that further extend into the target gene; ensuring specific amplification while covering a large number of genes with the same primer (Tables 1, 2).

**Table 1:** Sequence of the primers used to amplify and fuse the heavy variable chain genes into an scFv phage display library.

VH family	Primer	Nucleotide sequence
Family 1	MP20877	AAGGCCCAGCCGGCCATGGCCGAAGTCCAGCTGCAGCAGT
	MP20878	AAGGCCCAGCCGGCCATGGCCCAGGTCCAGCTGCAGCAGTC
	MP20879	AAGGCCCAGCCGGCCATGGCCCAGGTCCAGYTGCAGCAGTCTG
	MP20880	AAGGCCCAGCCGGCCATGGCCGAGGTAAAGCTGCARCAGTCTGGAG
	MP20881	AAGGCCCAGCCGGCCATGGCCCAGGTYAAGCTGCWGCAGTCTGG
	MP20882	AAGGCCCAGCCGGCCATGGCCCAGGTACAGCTGCAGCARTCTGG
	MP20883	AAGGCCCAGCCGGCCATGGCCCAGGTCCAGTTGCAGCARTCTGG
	MP20884	AAGGCCCAGCCGGCCATGGCCCAGGTCCAGCTGCAKCAGT
	MP20885	AAGGCCCAGCCGGCCATGGCCGARGTTCAWCTGCARCAGTCTGGG
	MP20886	AAGGCCCAGCCGGCCATGGCCGGAGTMCWGCTGCAGMAGTC
Family 2	MP20887	AAGGCCCAGCCGGCCATGGCCCAAGTGCARCTRAAGGAGTCAGGACC
	MP20888	AAGGCCCAGCCGGCCATGGCCCAGGTGCAGCTGAAGGAGACAGGACC
	MP20889	AAGGCCCAGCCGGCCATGGCCCAAGTGCAGTGGAAGGAGTCAGG
	MP20890	AAGGCCCAGCCGGCCATGGCCCAGGTGCAGCTCAAGGAGTCAG
Family 3	MP20891	AAGGCCCAGCCGGCCATGGCCGAGGTGCAGMTTCAGGAGTCAGG
	MP20892	AAGGCCCAGCCGGCCATGGCCCAGGTGAATCTTCAGGAGTCAGGACC
Family 4	MP20893	AAGGCCCAGCCGGCCATGGCCGAGGTGAARCTTGTCGAGTCTGGAGG
Family 5	MP20894	AAGGCCCAGCCGGCCATGGCCGAGGTGCAGCTGGTGGAGTC
	MP20895	AAGGCCCAGCCGGCCATGGCCGAGGTGCAGCTRGTGGAGWCTG
	MP20896	AAGGCCCAGCCGGCCATGGCCGAGGTACAGCTGGTKGAGTCTGG
	MP20897	AAGGCCCAGCCGGCCATGGCCGARGTGAAGCTGGTRGAGTCTGGG
	MP20898	AAGGCCCAGCCGGCCATGGCCGAGTCTGGGGGAGGATTAGTACAGC
Family 6	MP20899	AAGGCCCAGCCGGCCATGGCCGAGGTGAAACTGGAGGAATCTGGG
	MP20900	AAGGCCCAGCCGGCCATGGCCGAGGTGCAGCTTGTAGAGACAGG
Family 8	MP20901	AAGGCCCAGCCGGCCATGGCCCAGGTTACTCTGAAAGAGTCTGGTCC
Family 9	MP20902	AAGGCCCAGCCGGCCATGGCCCAGATYCAGTTGGTACAGTCTGGACCTG
Family 10	MP20903	AAGGCCCAGCCGGCCATGGCCGAGGTGCAGCTTGTTGAGTCTGG
Family 11	MP20904	AAGGCCCAGCCGGCCATGGCCGAAGYACAGCTGGTGGAGTCTGG
	MP20905	AAGGCCCAGCCGGCCATGGCCGAAGTGAAGCTGGTGGARTCTGGAG
Joining region (HJ)	MP20906	CCAGAGCCACCTCCGCCTGAACCGCCTCCACCTGAGGACACGGTGACCATGG
	MP20907	CCAGAGCCACCTCCGCCTGAACCGCCTCCACCTGAGGAGACTGTGACCATGACTCCT
	MP20908	CCAGAGCCACCTCCGCCTGAACCGCCTCCACCTGAAGAGACAGTGACCAGAGTGCC
	MP20909	CCAGAGCCACCTCCGCCTGAACCGCCTCCACCTGAGGAGACAGTGACTGAAGCTCC
Overlap extension PCR	VH outer primer	TAGATAGATTAAAGGCCCAGCCGGCCATG

The table includes forward and reverse primer specific for different genes of the heavy variable chain families and joining regions of *Rattus norvegicus* germline sequences. An outer forward primer was used for the overlap extension PCR with the kappa variable chains products to generate the scFv phagemid library. The primer may include an ambiguous DNA nucleotide (Y, R, W, S, K and M) to increase the coverage for families containing large number of subgroups while only 1 or 2 primers were required for smaller families. Four reverse primer were designed to cover all the joining regions of the VH.

**Table 2:** Sequence of the primers used to amplify and fuse the kappa light variable chain genes into an scFv phage display library.

VLK family	Primer	Nucleotide sequence
Family 1	MP20913	GGCGGAGGTGGCTCTGGCGGTGGCGGATCGGATGTTGTG + ATGACACAAACTCC
	MP20914	GGCGGAGGTGGCTCTGGCGGTGGCGGATCGGATGTTGTGTTG + ACACAAACTC
	MP20915	GGCGGAGGTGGCTCTGGCGGTGGCGGATCGGATGTTGTGATGACCCAGACACCAC
	MP20916	GGCGGAGGTGGCTCTGGCGGTGGCGGATCGGATATTGTG + ATGACMCAGACTCC
	MP20917	GGCGGAGGTGGCTCTGGCGGTGGCGGATCGGATGTTGTGMTGACCCAGACTCCA
Family 2	MP20918	GGCGGAGGTGGCTCTGGCGGTGGCGGATCGGATAT + TGTGATGACTCAAGCTCC
	MP20919	GGCGGAGGTGGCTCTGGCGGTGGCGGATCGGATATTGTGATGACCCAGGGTGCAC
	MP20920	GGCGGAGGTGGCTCTGGCGGTGGCGGATCGGATATCATGATGACTCAGTCTCCCCTCTC
Family 3	MP20921	GGCGGAGGTGGCTCTGGCGGTGGCGGATCGGACATTGTGCTGACCCAGTCTCC
	MP20922	GGCGGAGGTGGCTCTGGCGGTGGCGGATCGGACATTGTCTTGACCCAGTCTCCTG
	MP20923	GGCGGAGGTGGCTCTGGCGGTGGCGGATCGGACACTGTRCTGACCCAGTCTCC
Family 4	MP20924	GGCGGAGGTGGCTCTGGCGGTGGCGGATCGGAAATTGTGCTCACTCAGTCTCCAACAAC
	MP20925	GGCGGAGGTGGCTCTGGCGGTGGCGGATCGGAA + ATTGTGCTCAYCCAGTCTC
	MP20926	GGCGGAGGTGGCTCTGGCGGTGGCGGATCGGATAATGTGCTCCCTCAGTCTCCAAC
	MP20927	GGCGGAGGTGGCTCTGGCGGTGGCGGATCGGAAACTGTGCTCACTCAATCTCCAACC
	MP20928	GGCGGAGGTGGCTCTGGCGGTGGCGGATCGGCTATTGTTCTCAACCAGTCTCCATCCATC
	MP20929	GGCGGAGGTGGCTCTGGCGGTGGCGGATCGGAAAT + TATACTCACCCAGTCTCC
Family 5	MP20930	GGCGGAGGTGGCTCTGGCGGTGGCGGATCGGACATCGTGCTRACTCAGTCTCCA
	MP20931	GGCGGAGGTGGCTCTGGCGGTGGCGGATCGGACRTTGTGYTGACTCAGTCTCCAGC
Family 6	MP20932	GGCGGAGGTGGCTCTGGCGGTGGCGGATCGGACAYTGTGATGACCCAGTCTCC
	MP20933	GGCGGAGGTGGCTCTGGCGGTGGCGGATCGAACAYTGTGATGACYCAGTCTCCCA
Family 7	MP20934	GGCGGAGGTGGCTCTGGCGGTGGCGGATCGAATGTCATGATGACCCAGTCTCCAAC
Family 8	MP20935	GGCGGAGGTGGCTCTGGCGGTGGCGGATCGGACAT + TGTGATRACCCARTCT
	MP20936	GGCGGAGGTGGCTCTGGCGGTGGCGGATCGGACATTGCGATAACCCAGTCTCC
	MP20937	GGCGGAGGTGGCTCTGGCGGTGGCGGATCGGACAT + TKTGATGAC + CCAG
	MP20938	GGCGGAGGTGGCTCTGGCGGTGGCGGATCGGACATTTTGATAAACCAGTCTCCAGCCTC
Family 9	MP20939	GGCGGAGGTGGCTCTGGCGGTGGCGGATCGCAGATCACGCTCACCCAGCAAG
Family 10	MP20940	GGCGGAGGTGGCTCTGGCGGTGGCGGATCGGACATCCAGWTGACCCAGTCTCCATC
	MP20941	GGCGGAGGTGGCTCTGGCGGTGGCGGATCGGACATC + CAGWTKACCCAG
Family 12	MP20942	GGCGGAGGTGGCTCTGGCGGTGGCGGATCGGACATCCA + GATGACACAGTCTCC
	MP20943	GGCGGAGGTGGCTCTGGCGGTGGCGGATCGGATATCCRGATG + ACWCAGTCTCC
Family 14	MP20944	GGCGGAGGTGGCTCTGGCGGTGGCGGATCGGACATCCAGATGACCCAGTCTCC
	MP20945	GGCGGAGGTGGCTCTGGCGGTGGCGGATCGGACATTCAGATGACCCAGTCTCCATCC
	MP20946	GGCGGAGGTGGCTCTGGCGGTGGCGGATCGGACATTCAGATGACSCAGKCTYCATC
Family 15 to 20	MP20947	GGCGGAGGTGGCTCTGGCGGTGGCGGATCGGATRTCCAGATG + ACMCAGTC
	MP20948	GGCGGAGGTGGCTCTGGCGGTGGCGGATCGGAAACTACTGTGACCCAGTCTCCAGC
	MP20949	GGCGGAGGTGGCTCTGGCGGTGGCGGATCGACTGGAGAAACAACACAGTCTCCAGC
	MP20950	GGCGGAGGTGGCTCTGGCGGTGGCGGATCGGACATTAGGATGACTCAGACTCCAGC
	MP20951	GGCGGAGGTGGCTCTGGCGGTGGCGGATCGGACATCCACATGACTCAGAACCCAG
Joining region (KJ)	MP23454	TGCACGCTGCTAGATATGAGGCACTGCGGCCGCGTTTCAATTCCAGCTTGGTGCCTCC
	MP23455	TGCACGCTGCTAGATATGAGGCACTGCGGCCGCGTTTCAGTTCCAGCTTGGTCCCAG
	MP23456	TGCACGCTGCTAGATATGAGGCACTGCGGCCGCGTTTTATTTCCAGTCTGGTCTCATCACTG
	MP23457	TGCACGCTGCTAGATATGAGGCACTGCGGCCGCGTTTTATTTCCAACTTCGTCCCTGAGCC
	MP23458	TGCACGCTGCTAGATATGAGGCACTGCGGCCGCGTTTGATCTCCAGCTTGGTCCCAGAAC
Overlap extension PCR	VLK outer primer	TGCACGCTGCTAGATATGAGGCAC

The table includes forward and reverse primer specific for different genes of the kappa light chain families and joining regions of *Rattus norvegicus* germline sequences. An outer reverse primer was used for the overlap extension PCR for the assembly as scFv phagemid library. The primer may include an ambiguous DNA nucleotide (Y, R, W, S, K and M) and Locked Nucleic Acids (LNAs) were incorporated in specific conserved residues of the variable chains’ genes facilitating the generation of short primers to avoid extension into the frame, minimizing mismatches in other regions of the primer. Five reverse primer were designed to cover all the joining regions of the VLK.

For VH regions, on average each primer amplified 23 variable genes, although smaller families required 1 or 2 primers (Supplementary Table S1). The kappa light chain genes contain a higher number of families, but a smaller number of different subgroups (Supplementary Table S2). This allowed the coverage of each family with fewer primers per family. In this instance, an average of 19 subgroups were covered per primer. We designed 4 reverse primers for VH [annealing to the heavy chain joining regions (HJ)] and 5 reverse primers for the VLK [annealing to the kappa chain joining regions (KJ)]. Together these primers cover all the possible joining regions in rat VH and VLK.

The primer tail at the 5′ end of the VH gene and the 3′ end of the VLK gene contained the annealing sites for the nested PCR outer primers and *SfiI/NotI* restriction sites for cloning into the phagemid vector (pHEN1^19^). The tails at the 3′ end of the VH and the 5′ end of the VLK included the serine-glycine linker sequence (3xGGGGS) as overlapping regions. The primary PCR amplification of VH and VLK chains incorporated the outer tail regions. Overlap extension PCR created a single amplicon encoding an scFv in the VH-VLK orientation separated by a serine-glycine linker sequence.

### Primer set effectively amplifies VH and VL genes without PCR-biases

We first sought to better understand the VH/VLK usage in rats, since this has not been well studied. We then could determine if our novel primer set result in biased amplification of certain VH/VLK families. Deep-sequencing of VH/VLK genes from 5′RACE and primer set amplified cDNA were studied and compared; cDNA was isolated from three wild type naïve, antigen unchallenged, *Wistar* rats. The material was used as template for both an Ig specific 5′RACE PCR and for primer set amplification and sequenced using the Illumina MiSeq Next-generation sequencing (NGS) platform.

**Figure 1:**
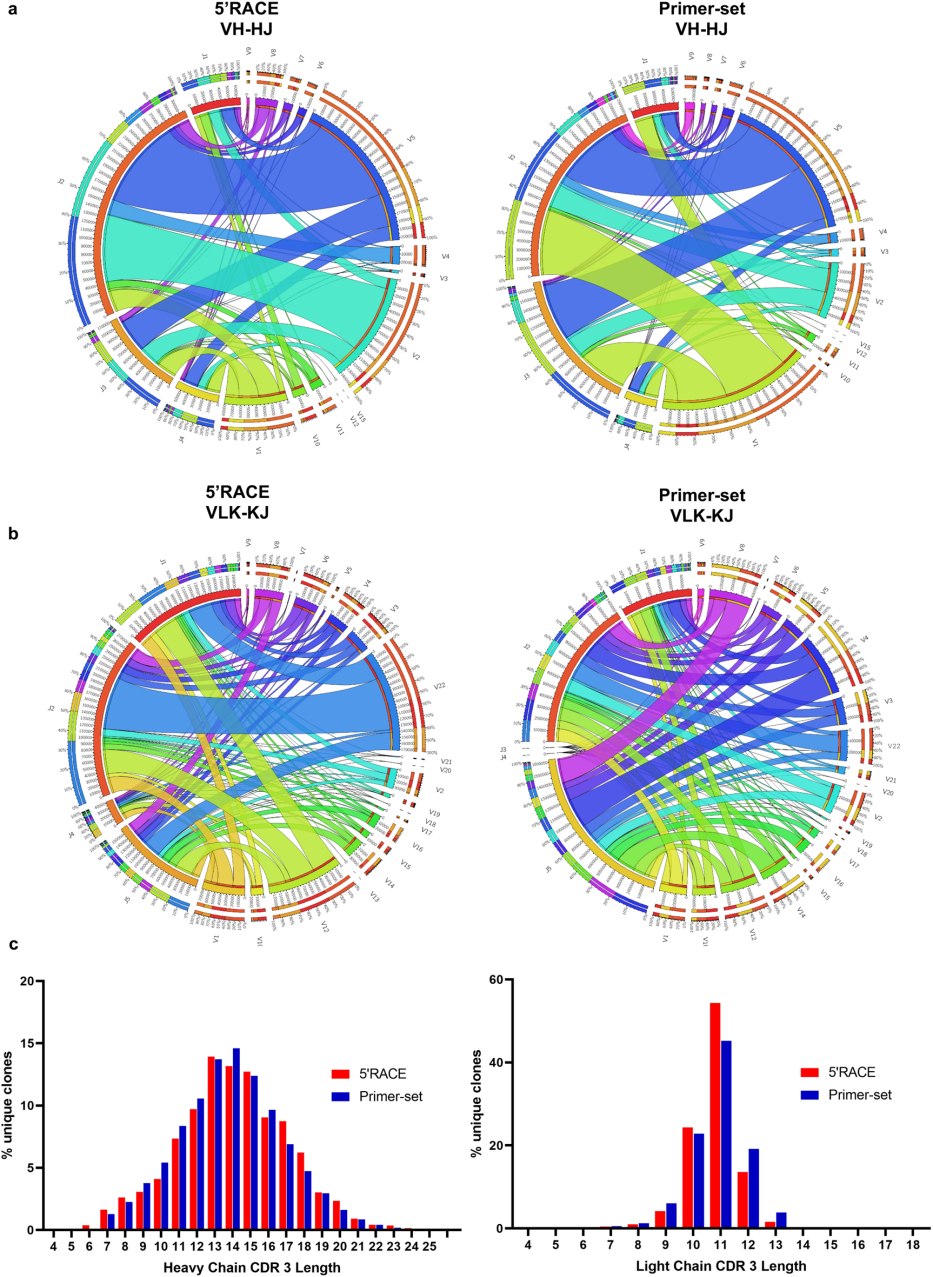
NGS analysis of the VH and VLK genes in naïve rats. Chord diagram representation of the V and J genes frequency and their respective pairing; analysis of the 5′RACE-amplified cDNA(left) and primer set amplified (right) for VH (**a**) and VLK (**b**) genes. The diagrams are color-coded showing the pairing network of the joining regions with each variable gene family, the size of the inner segments correlates with the number of sequences identified for each gene. The frequency of unique CDR3 sequences with different nucleotide lengths in the heavy chain and kappa light chain genes are shown as histograms (**c**) for both the VH (left) and VLK (right).

Analysis of the VH and VLK usage in naïve rat following 5′ RACE showed that VH family 5 was the most represented (39%) followed by family 2 and 1 with 28 and 16% respectively. The other families were all present at a lower frequency (1–4%) while family 12 and 15 were the least represented with a frequency of less than 0.1%. Analysis of VLK usage showed that family 22 and 12 and 1 were the most represented (28%, 20%, 12% of the total VLK usage respectively) while the majority of VLK families were present at similar frequencies between 3 to 5%. Families, 5, 9, 17, 19 and 21 were present at less than 1% and family 7, 13, 18 and 20 were less than 0.1% (Fig. 1).

In order to determine whether our primer set was able to amplify the rat VH/VLK repertoire in an unbiased fashion, we compared the repertoire from 5′RACE described above with that amplified by our primer set, again in the context of unchallenged naïve rats. The relative frequency of variable heavy (VH) and joining (HJ) genes was similar in both primer and 5′RACE amplified sets (Fig. 1a). In the 5′ RACE amplified dataset 55% of the VH genes were paired with J2 the next most frequently used J region was J3 while a similar proportion of J1 and J4 genes were used. The V-J pairing distribution was maintained after primer set amplification in which J2 remained predominantly used (49%), followed by J3 (33%). Thus, the frequency of the VH family usage was preserved before and after V region specific PCR.

The kappa light chain (VLK) samples were similarly analysed. Here we observed that the V-J pairing pattern was comparable in both 5′RACE and primer set amplified products (Fig. 1b). For example, the VLK22-KJ2 pairing frequency was observed at 55% and 50% in the 5′RACE and primer amplified samples respectively. This again suggested that the PCR amplification using gene specific primers did not bias the repertoire towards specific clones. The general frequency of the usage for each kappa V chain family and J genes did not show differences in the two samples.

The PCR products obtained after V region specific amplifications demonstrated a similar representation of VLK families as the 5′RACE sample. IgVLK7, IgVLK13, IgVLK18, IgVLK20, IgVLK21 were present at low frequency in the pre-PCR sample and were observed at a similar proportion post-amplification with the primer set. A notable omission in the primer set sample was the absence of IgVLK13 which had a low representation in the 5′RACE dataset and a reduction in the frequency of the J4 region in the kappa light chain pairing. The larger proportion of J5-assigned sequences suggest that the J5 reverse primer, which shares homology with J4, may have preferentially amplified this region over the J4. Overall, both the frequency of the gene-usage and the V-J pairing suggest that PCR amplification using this primer set did not skew the rat’s immune repertoire, but enabled generation of V-J products as close as possible to the originally repertoire present in these rats.

As additional confirmation, we have looked at the frequency distribution of unique CDR3 sequences within both the VH and VLK genes in the naïve rat data sets. The percentage unique clones containing CDR3 sequences of different length was almost identical in 5′RACE and primer set amplified genes for both heavy and light chain (Fig. 1c). Gaussian analysis of the data indicated a mean CDR3 length of 14.09 and 13.78 for the heavy chain and 10.87 and 10.94 for the light chain, in 5′RACE and primer amplified data sets respectively (Supplementary Fig. S1). This data supports the lack of bias introduced by the PCR amplification of the variable genes and demonstrates that the overall diversity is faithfully represented with our primer-set.

### Generation and selection of an scFv immune phage library from CD160-vaccinated rats

The human CD160 open reading frame was cloned into the pVAC2 expression vector (pVAC2.CD160). Expression was confirmed by transient transfection in HEK293T cells and staining with the commercial BY55 antibody. Three *Wistar* rats were genetically vaccinated using the plasmid pVAC2 encoding CD160. Twenty-one days post vaccination, serum-conversion was observed in all three rats (Supplementary Fig. S2). CD160 was also cloned into the retroviral vector SFG and SupT1 cells expressing high levels of CD160 were generated (Supplementary Fig. S3).

Pooled cDNA from the three vaccinated rats was used as template with 68 individual PCR reactions (29 for the VH and 39 for VLK primers). Reverse primers for the joining region were used in an equimolar mixture for each individual forward primer PCR reaction. (Supplementary Fig. S4). A notable feature was the presence of the VH family 3 which was sequenced at low frequency (0.4%) from 5′RACE amplicon in naïve rats. In the case of CD160-immunised animals this region was successfully amplified with only one of the two primers designed to anneal to this region (MP20891). After overlap extension PCR, the approximately 800 bp scFv insert was subsequently cloned in the phagemid vector and displayed on the phage surface for biopanning.

**Figure 2:**
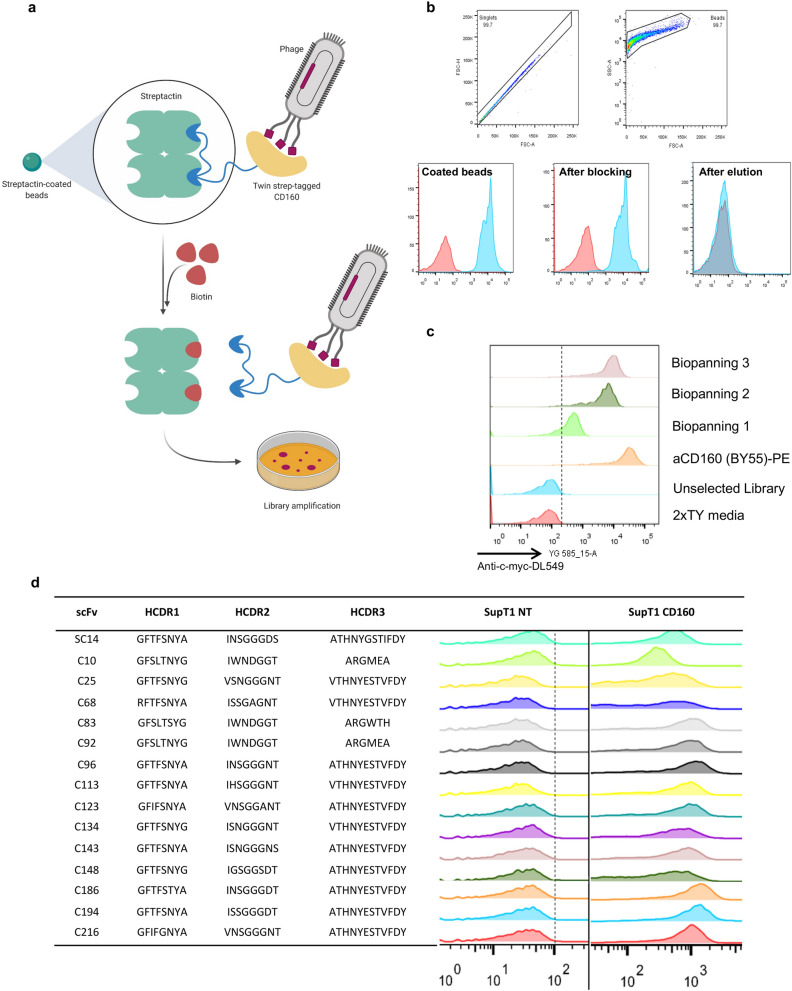
Biopanning of the scFv phage display library on Streptactin magnetic beads. (**a**) Schematic representation of the biopanning process employing the StreptagII-Streptactin system. CD160 fused with Twin streptag was capture directly from cell supernatant on the surface of Streptactin magnetic beads. Phages were incubated with the antigen’ coated beads and bound phages were subsequently recovered by eluting with a 30 mM biotin solution and used to amplify the library for the next biopanning round. (**b**) The process of coating and elution of the beads throughout the biopanning process was assessed by flow cytometry analysis. These were stained during the several steps of the procedure (coating, blocking with 3%BSA and elution) using commercial anti CD160-PE. (**c**) Enrichment of the phage library for CD160 was determined after three rounds of biopanning on beads. The bacterial library from the different rounds of selection was induced to express the soluble scFv fragment in the supernatant. This was used to stain SupT1 cells overexpressing human CD160, due to the presence of a myc-tag at the C-terminus of productive scFvs we were able to detect binding using anti myc antibody. 2xTY media + anti myc-tag-DL549 and the commercial anti-CD160-PE Ab were used as controls. (**d**) The 15 individual bacterial colonies identified in the third round of biopanning carrying unique combination of CDR1,2 and 3 were screened by against SupT1 CD160 positive and negative cells (NT).

We displayed the human GPI-anchored protein CD160 on Strep-Tactin magnetic beads. This facilitated direct capture on to beads from the supernatant of transfected HEK293T cells and convenient elution with biotin (Fig. 2a). To confirm quality of the CD160 protein from the transfected HEK293T, we prepared it to 95% purity, analysed it by size exclusion chromatography (SEC) and differential scanning fluorimetry (DSF), and compared it with different sources of CD160 protein (Supplementary Fig. S5). The success of the biopanning process was assessed by flow cytometry staining of the beads at the various stages using an anti-CD160 antibody (Fig. 2b). Three sequential biopanning rounds of the phage-scFv library were conducted using the CD160-Strep-Tactin magnetic beads; enrichment was tested after each round of selection. To increase the likelihood of finding functional binders against the native conformation of the target antigen, we screened individual clones using flow cytometry. Successful enrichment was assessed using IPTG-induced bacterial supernatant to stain CD160 positive SupT1 cells (Fig. 2c). A clear binding of the supernatant was observable in selection rounds 2 and 3, but a small enrichment is also evident in round one, highlighting the presence of specific binders after a single round of phage selection. Screening clones derived from individual bacterial colonies identified 15 unique scFvs that specifically bind CD160 positive cells (Fig. 2d).

### Characterization of identified binders revealed high affinity kinetic clones

**Figure 3:**
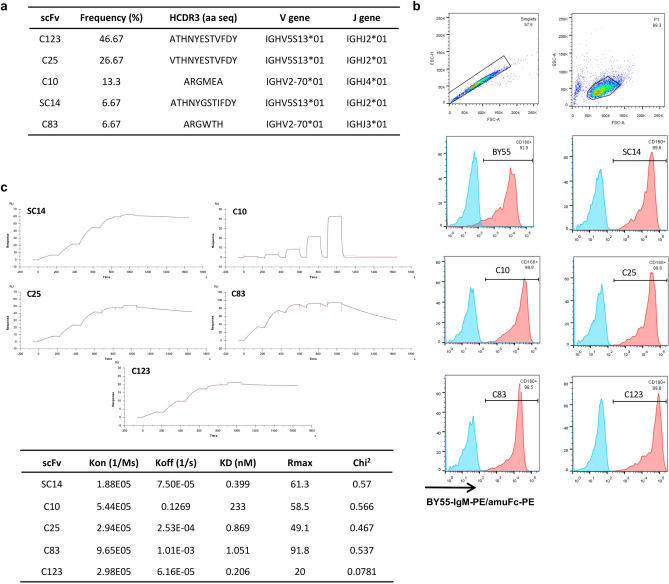
Characterization of the monoclonal scFv identified in the biopanning. (**a**) Alignment to the IMGT database of *Rattus norvegicus* germline variable sequence showed rearrange sequences originated from family 2 and 5 of the VH genes. We identified five different HCDR3 in which three were represented in multiple clones and three occurring only once. (**b**) Flow cytometry analysis of the five selected HCDR3 expressed secreted as chimeric scFv fused with mIgG2aFc. Supernatant from transfected HEK293T cells was used to stain SupT1 negative and SupT1 CD160 positive cells; the binding was detected with a secondary anti-mouseIgGFc-PE and anti CD160-PE (BY55) was used as positive control. (**c**) The affinity of these five scFv was determined using Biacore T200 technology. Individual scFvs were captured on a CM5 chip and CD160 was injected as analyte at 5 different concentration (3.70 nM, 11.11 nM, 33.33, nM, 100 nM and 300 nM) in a single-cycle kinetic study. The double-reference subtracted sensorgrams fitted with the 1:1 Langmuir binding model; kinetic and model fitting data are reported in the table.

Alignment of the VH genes to the IMGT database of rat germline variable genes revealed that the 15 unique scFv clones which contained 5 different HCDR3 originating from rearranged germline family V2 and V5. Two of these CDR3s were found to account for 46.67% and 26.67% of the total diversity (Fig. 3a).

Five binders (SC14, C10, C25, C83, C123) carrying unique CDR3s were studied further as recombinant chimeric rat scFv/mouse IgG2aFc antibodies. Flow cytometric analysis determined that the recombinant antibodies selectively bound cell lines engineered to express CD160 (Fig. 3b).

The kinetic profile of the scFvs was studied by SPR using purified CD160 as the analyte. We found a range of high affinity scFvs (Fig. 3c). SC14, C25 and C123 were the highest affinity with KD values of 0.399, 0.869 and 0.206 nM respectively. These clones all exhibited rapid association and slow dissociation kinetics as expected for high affinity binding antibodies, (k_a_ = 1.8–5.4 × 10^5^, k_d_ = 2.53 × 10^–4^–7.50 × 10^–5^). The KD values of clones C10 and C83 were 233 nM and 1.051 nM. Clone C83 demonstrated a rapid association (k_a_ 9.65 × 10^5^) and intermediate dissociation rate (k_d_ = 1.01 × 10^–3^). Interestingly, despite C10 and C83 showing equivalent binding in flow cytometry experiments, clone C10 demonstrated a very rapid dissociation rate (k_d_ = 0.1269) and the binding was likely compensated by a rapid association rate (k_a_ = 5.44 × 10^5^). The range of kinetics observed in the clones suggest that the binders selected from the library will have utility in a wide range of applications.

### CAR engineered T cells were functional and able to specifically kill CD160 positive cells

We next sought to demonstrate the therapeutic potential of CD160 binders. For this we converted the 5 different CD160 scFvs into CAR format. A third generation CAR construct with CD28 and OX40 costimulatory endodomains^20^ and with a CD8STK as spacer moiety^21^ was selected as the format for CAR comparison.

**Figure 4:**
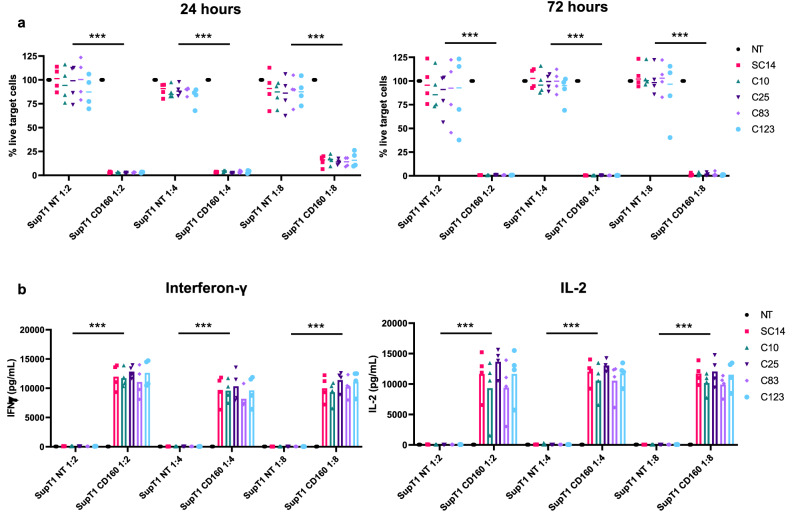
Functional assays as chimeric antigen receptor. Four different healthy donors PBMCs were transduced with lentivirus to express the five different scFvs fused in a third generation (CD28-OX40ζ) CAR structure and tested in a cytotoxic assay. T cells were incubated with SupT1 CD160 positive and negative cells (NT) at different effector to target ratio (1:2, 1:4 and 1:8). The residual number of target cells for each donor was normalized against non-engineered PBMCs and all the five CARs demonstrated excellent killing at both 24 h and 72 h (**a**). Total cytokine production was assessed at 72 h and showed high levels of interferon-gamma (IFN-γ) and IL-2 (**b**) with no background on target-negative cells. The mean from each donor was plotted individually and analysed using two-way ANOVA with Dunnett’s post-test for comparison between CARs within the same target group. ***p < 0.0001.

Normal donor, peripheral blood T cells were transduced to express CAR constructs using gamma retroviral vectors. Transduced T cells were then co-cultured at different effector to target ratios (E:T) with either SupT1 cells (which do not express CD160) and SupT1 cells engineered to express CD160. One day post co-culture we observed already a drastic control in the growth of the SupT1 cells expressing CD160, with complete killing of the CD160 target cells by 72 h (Fig. 4a) at all the E:T tested. Furthermore, the 5 different CARs showed no non-specific killing with complete recovery of the NT target cells at both time point.

The 5 different CD160 CARs were further evaluated for the secretion of pro-inflammatory cytokines interferon-gamma (IFN-γ) and Interleukin-2 (IL-2) at 72 h. All CARs tested showed high levels of cytokine production (Fig. 4b). No background cytokine release was observed to SupT1 cells. No differences were observed at any of the effector to target ratio tested, suggesting equal potency of all CARs generated. An interesting feature of all CARs tested was the high levels of IL2 secretion observed. This particular feature could be beneficial in the improvement of CAR T cells persistence in vivo.

## Discussion

Phage display has been successful in generating a large number of antibodies used in a variety of application including therapeutics^22^. Immune phage libraries combine advantages of both phage display and hybridoma technology. The in vivo selective expansion of high affinity B cell clones after immunization allows use of a small phage library and typically, multiple high affinity binders can be identified after limited rounds of bio-panning^23,24^.

Immune libraries are typically derived from mice whose immune repertoire has been exhaustively studied^25^. Rats are rarely used for construction of such phage libraries, despite having advantages over mice as a host species to generate antibodies. For example, the propensity of rats to undergo rapid serum-conversion typically means that fewer animals need to be vaccinated^7^. Furthermore, being larger than mice, a tenfold greater amount of serum can typically be extracted, and larger secondary lymphoid organs allow the isolation of a greater number of lymphoid tissues. The use of rats as vaccination host results in non-cross-reactive antibodies and species-specific epitopes, distinct from those raised in mice^26–28^.

Primers designed for the amplification of mouse immunoglobulins^29,30^ have been successfully applied for the generation of scFv based phage libraries from immunized sources^31,32^. However, few primer sets that specifically amplify immunoglobulin variable regions from rats have been described. In 2008, Sepulveda and Shoemaker^6^ compared rat EST sequences and mouse VL/VH coding sequences and designed a primer set which amplifies 65 heavy and 77 light chains-predicted variable domains. From the curated IMGT database, we currently know that in spite of the close similarity to mouse, rats have a very high number of functional variable genes in the genome (232 VH and 164VLK) with IGVH1, IGVH2 and IGVH5^33^ being the most abundant families. Sepulveda’s primer set only covers family 1 and 2, lacking coverage of the highly abundant family IGVH5 and other families such as IGVH8 and IGVH11. The Sepulveda primer set covers only IGVLK-IGVLK6 families, which is only a small proportion of possible Kappa light chain variable sequences.

In order to expand the possibilities of using phage display-based methods for antibody discovery from immunized rats, we have utilised the curated IMGT database to design a set of primers that covers all currently known VH and VLK germline sequences in rats^16^. We used two strategies to exploit the similarity shared within each family at the start of the VH genes to minimize the numbers of primers: First, we incorporated up to 3 ambiguous nucleotides in each primer and secondly, we incorporated LNAs to shorten primers within highly homologous regions. This allowed us to cover all the families with a small number of primers (29 for VH and 39 for VLK) while fully covering the most represented families, such as IGVH1, IGVH2 and IGVH5^33^. Indeed, these were the most expressed families in rats as shown by NGS, confirming that the design of the primer set specifically captures the diversity of rat’s immunoglobulins response.

Because the rat’s immunoglobulin repertoire is relatively unstudied, we first used NGS sequencing to investigate the family’s usage in rats. This confirmed the IMGT annotation^33^ with family 5 being the most represented VH family followed by an equal frequency of family 1 and 2, while for VLK we identified family 22 and 12 as the most commonly used genes in naïve rats. We compared these data with NGS of pooled VH and VLK amplified with our primer set to determine if our primer library resulted in any bias. Families containing fewer subgroups were also identified at lower frequencies and were covered proportionally in both the data sets. A similar profile of J region associated with each VH gene also confirms lack of bias introduced by the PCR with our primer set. Finally. the analysis of the CDR3 distribution in both the 5′RACE and primer set-amplified products demonstrated an almost identical pattern of diverse CDR3 length in both the VH and VLK genes, again confirming faithful amplification.

To validate the performance of the primer set we used CD160 as a test antigen for the generation of a rat immune library. CD160 is a small protein expressed either as a GPI-anchored protein, with physiological expression restricted to NK cells and a subset of T cells. CD160 is a promising target for a number of clinical applications. The protein is expressed in neovasculature^13^, and has the potential to be used for vasculature targeting. It has been found in exhausted HIV-specific CD8+ T cells^34^ and aberrantly expressed on B cell lymphoproliferative disorders^14^.

We generated a phage display immune library for human CD160 from DNA immunized rats. We selected the immune phage display library with CD160 fused to the Strep-tag II peptide at the carboxy-terminus of the antigen. This construct facilitated direct capture to magnetic beads from the supernatant of transfected cells, avoiding the necessity of purification, biotinylation or passive absorption of the target molecule. Further, exploiting the higher affinity of biotin to Strep-Tactin compared with Strep-tag II, the addition of biotin allowed the gentle desorbing on phage from the beads through competitive elution. Sequence analysis identified fifteen unique scFvs with five different HCDR3 from either family 2 or 5 of the rat germline VH genes. Furthermore, characterisation of the obtained binders showed specific binding to the target with binding affinities from the mid-nanomolar to picomolar range.

CAR T cells have demonstrated success in the treatment of lymphoid malignancies targeting CD19, and more recently BCMA^35,36^. To demonstrate the utility of the discovered scFv, we derived CARs from 5 of the scFv with unique HCDR3. Primary human T cells engineered to express these CARs showed specific cytokine release and cytotoxicity and in response to cells expressing CD160. Interestingly, there was limited difference in the cytotoxic capacity or secretion of cytokine despite measurable differences in the binding affinities of the different binders.

Here, we describe a method of antibody isolation using phage display libraries based on a novel set of primers which amplifies the entire rat variable VH and VLK repertoires. We demonstrated the utility of such libraries in combination with genetic immunization and a simple display technique to generate antibodies, with a range of affinities to the target CD160. We anticipate this primer set will be of use in generating immune libraries from rats, in addition to other applications such as single B-cell antibody discovery methods. Finally, we hope that the overall methodology described here, combining immunization and panning strategy, opens up rapid and simple antibody discovery to laboratories with limited experience and resources for antibody engineering for CAR T and other applications.

## Materials and methods

### Rat immunizations, RNA extraction and cDNA synthesis

Three adult *Wistar* rats were immunized with the target human CD160 (accession number: NM_007053.3). Genetic vaccinations were carried out using DNA encoding coated gold nanoparticles at Aldevron, GmBH. Briefly, the gene coding for human CD160 was cloned into a pVAC2 vector for the rats vaccination. A GeneGun system was used to deliver plasmid coated gold nanoparticles intramuscularly. Homogenized lymph nodes and spleens from the vaccinated animals were preserved in RNA later (QIAGEN). RNA was extracted from 7 × 10^6^ lymph node cells using RNAeasy kit (QIAGEN). RNA from multiple aliquots of cells was pooled together for downstream RT-PCR. The cDNA reaction was performed with 7 µg of RNA using QuantiTect Reverse Transcription kit (QIAGEN). The success of the reaction and potential genomic contamination was assessed by PCR amplification followed by gel electrophoresis of the housekeeping gene GAPDH using a 1:5 working dilution of the cDNA.

All animals were used under protocols approved by UCL Animal Welfare and Ethical Review Body (AWERB) and authorization granted by the UK Home Office under the Animal Scientific Procedures Act 1986 (licence number: 12570).

### 5′ RACE amplication of Ig genes naïve rats

Three 8–10-week-old wild-type Wistar rats (2 female and 1 male) were purchased from Charles River, UK. The animals were sacrificed and RNA extracted from homogenised spleens. Applying the Simple Method for Amplifying RNA Targets (SMART)^37^, Superscript II Reverse Transcriptase (Invitrogen) was used to generate cDNA; the reaction was primed to incorporate a specific tail for subsequent PCR. The resulting cDNA from each animal was then pooled and used to amplify the total VH and VLK gene set. The PCR was performed at 68 °C using KOD polymerase (Thermo Scientific) and a forward primer (5-cgacgtggactatccatgaacgca-3′) specific for the tail in the cDNA with a reverse primer specific for either the CH1 portion of the rat IgG’s constant region (5-ccagactgcaggacagctgg-3′) or the kappa constant region (5′-atgatgtcttatgaacaacctcacaggtatagag-3′). Successful amplification was confirmed on an agarose gel by the presence of product between 600 and 700 bp. The DNA was subsequently purified by ethanol precipitation and resuspended in 20 μl of nuclease-free dH_2_0. The amplified fragments for VH and VLK chains were used for NGS sequencing.

### NGS sequencing and analysis

The total pool of VH and VLK products were sequenced using an Illumina MiSeq platform with 2 × 300 bp paired-end configuration (GENEWIZ). A quality FASTAQ report was delivered and used as input files in sequence reconstruction. The software for adaptative immune profiling MiXCR^38^ was employed to overlap the paired-end sequencing and the rat’s germline database obtained from IgBlast^39^ was used as reference sequences for the alignment of the V, D and J segments. Output of each processing stage was converted to tab-delimited text files for manual inspection. Gene segments were identified using IMGT’s nomenclature, including functional and open reading frame-defined gene segments. All productive VH and VLK segments were analysed and the percentage of usage was determined by dividing sequencing reads corresponding to each gene segment by the total number of gene segments. V(D)J pairing was only assessed from productive VH and VLK segments. Undetermined or unproductive sequences were excluded. Total counts from V-J pairings for heavy- and light-chains were tabulated and chord diagrams were generated using Circos Online^40^.

### Amplification of VH and VLK chains

The primary amplification of the two variable chains was performed with oligonucleotides designed on the *Rattus norvegicus* germline sequences of heavy variable chain (VH) and kappa light chain (VLK) genes; primers were designed to have the annealing portion with a melting temperature around 60 °C (Table 1). A PCR master mix was prepared using High Fidelity Phusion polymerase and buffer (NEB) in a 50 µl reaction with 1 µl of a 25 nM working dilution of each primer and 1 µl of the cDNA pool. An individual reaction for each forward primer containing 1 µl of an equimolar pooled mix of the reverse primers was performed. The samples were heated at 98 °C for 2 min, followed by 35 cycles of 98 °C for 30 s, 60 °C for 40 s, and 72 °C for 40 s with a 10 min final extension at 72 °C. Each product at approximately 400 bp was purified from the agarose gel.

### Overlap extension PCR

The amplified primary PCR products of VH and VL were pooled together for the overlap extension reaction. The outer primers were design to anneal 5′ end of the VH gene and the 3′ end of the VL gene for fusing into scFv format linked by a (Gly_4_Ser)_3_ repeats as linker. The reaction mixture was amplified as above using 100 ng of each pool. After the initial denaturation for 2 min at 98 °C the PCR was cycled as follows: 98 °C for 30 s, 58 °C for 40 s and 72 °C for 1 min with a final extension at 72 °C for 10 min. The PCR product was run on agarose gel and the 800 bp band was extracted and purified using QIAquick PCR clean up kit (QIAGEN). The reaction was scaled up for the subsequent enzymatic digestion.

### Generation of the scFv library

1 µg of purified fused product was digested with SfiI and NotI restriction enzymes (NEB) and cloned in to the phagemid vector pHEN1. The generated ligation mixture of plasmid DNA was cleaned-up by ethanol precipitation and re-suspended in 80 µl of dH_2_O prior to the electroporation into TG1 *E. coli* bacteria (MicroPulser, Bio-Rad). The transformed bacteria were recovered in 2xTY medium at 37 °C shaking at 225 rpm for 1 h before spreading on a Bio-Assay Dish (Fisher Scientific) containing 200 ml of 2xTY-agar, 100 µg/ml ampicillin and 1% glucose (w/v). Following overnight incubation, the dish was harvested and the library re-suspended in 10 ml of 2xTY and 15% glycerol (v/v) for storage in 500 µl aliquots at − 80 °C. The successful generation and estimation of the library size was performed by counting the colonies plated on serially diluted 2xTY-agar/Amp/Gluc petri-dishes; a number of single colonies were grown in liquid culture and the plasmid DNA was screened by colony PCR using M13 primers that generated a ~ 1000 bp DNA in the presence of the full-length scFv insert (~ 800 bp). The library glycerol stock was then used to inoculate the starting culture of the phage production for the first biopanning round.

### Phage production

To produce phage-scFv particles for the biopanning steps, a 2xTY/Amp/Gluc starting culture (OD_600nm_: 0.1) was inoculated with the library glycerol stock and grown to OD_600nm_: 0.4–0.5, before infection with 100 µl of M13KO17 helper phage (NEB) followed by incubation for 40 min at 37 °C without shaking. The bacteria were then centrifuged and re-suspended in 100 ml 2xTY containing 100 µg/mL ampicillin, 50 µg/mL kanamycin without glucose overnight at 30 °C shaking at 225 rpm.

Next day, the culture was centrifuged at 3300×*g* for 20 min at 4 °C to remove bacterial cells, 18 ml of ice-cold 20% PEG-6000/2.5 M NaCl was added to the phage-containing supernatant and the solution was incubated 4 °C for 1 h. After centrifugation at 3300×*g* for 20 min at 4 °C, the supernatant was discarded and the phage-containing pellet was re-suspended in 1.5 ml of dH_2_O. The solution was then centrifuged at ≥ 14,000×*g* in a microfuge for 5 min to pellet any remaining bacterial cells and the supernatant was transferred into a clean 1.7 ml tube. 0.25 ml of ice-cold 20% PEG-6000/2.5 M NaCl added, the solution was incubated at 4 °C for 1 h then centrifuged at ≥ 14,000×*g* for 10 min; the supernatant was discarded and the final phage pellet was re-suspended in 1 ml of dH_2_O. To assess the titre of phage particles, 10 µl were kept aside for *E. coli* infection and the remaining solution was divided into two ~ 500 µl aliquots which were blocked with 1 ml of PBS, 4% BSA (w/v) for 1 h at room temperature and subsequently used for the rounds of biopanning.

### SDS-PAGE

CD160-containing supernatant from HEK293T cells and mouse IgG2a Fc purified protein were run by SDS-PAGE alongside a purified commercial source of CD160-His (Sino Biological). 1 µg of purified protein or 5 µl of supernatant were run on to a TGX 4–20% gel according to the manufacturer’s protocols (Bio-Rad). Samples were prepared as follows: 5 µL of sample loading buffer, 2 µL of reducing buffer (not included under non-reducing conditions), made up to 20 µL final volume with PBS. Following SDS-PAGE, the gel was covered with Coomassie R-250 stain solution (Sigma-Aldrich) and stained for 60 min with gentle agitation on a rotating plate shaker. Staining solution was decanted and the gel was destained (dH_2_O, methanol, and acetic acid in a ratio of 50/40/10 (v/v/v)) until the protein bands became visible. Images of the proteins gel were acquired using QuantStudio imager (GE Lifesciences).

### Differential scanning fluorimetry

Protein stability was analysed on a Prometheus NT.48 (NanoTemper Technologies GmbH, Munich, Germany) as previously described^41^.

### ELISA

Antigens (CD160-Fc HEK supernatant 1:10 dilution; CD160-Fc HEK purified 1 µg/ml, 0.5 µg/ml, 0.25 µg/ml; CD160-Fc CHO purified 1ug/ml, 0.5 µg/ml, 0.25 µg/ml; CD160-His Sino biologicals) were coated in Nunc Maxisorp plates in coating buffer (Biologend, 50 μl/well) O/N at 4C. Plate were washed four times with PBS 0.05% Tween20, and blocked in 2% BSA (200 µl) for 1 h at RT. Primary antibody (BY55-humanFc HEK supernatant) was incubated with 1:3 serial dilutions in PBS 0.5% BSA for 1 h at RT. Plates washed in PBS 0.05% Tween20 4 times and incubated with anti-human HRP (Jackson Immunotools) at 1:3000 dilutions in PBS 0.5% BSA for 1 h at RT. Plates washed four times with PBS 0.05% Tween20, developed using one step TMB ultra (Thermo) 45 μl and stopped with 1 M H_2_SO_4_ 45 µl. The absorbance of the plate was read at 450 nm and used to determine the EC50 of the different formats of CD160 protein.

### Biopanning on magnetic beads

The biopanning rounds were performed using Strep-Tactin type II (IBA Lifesciences) magnetic beads coated with the target of interest. Human CD160 was genetically engineered to be fused with a mouse IgG Fc and a Strep-tagII (WSHPQFEKGGGSGGGSGGSAWSHPQFEK) and cloned as a secreted protein into a SFG expression plasmid as described elsewhere^42^. HEK293T cells cultured in complete DMEM medium (Gibco) were transfected with 12.5 µg plasmid DNA using GeneJuice transfection reagent (MerckMillipore). After 48 h, the cell supernatant was harvested and 0.2 µm filtered prior to the coating of magnetic beads. 5 µl of beads suspension were incubated with 1.5 ml of filtered cell supernatant for 1 h at room temperature shaking. Supernatant from non-transfected HEK293T cells was used to coat magnetic beads as a negative control during phage selection. The beads were magnetized and washed three times with PBS. The presence of CD160-mIgGFc-Strep-tag II fusion protein on the beads was assessed by flow cytometry of a representative aliquot of NT-supernatant coated beads and CD160-coated beads stained with anti-CD160 antibody (BY55-PE, BD Pharmigen). 20 µl of beads coated with NT-supernatant and CD160-supernatant were respectively incubated with the blocked phage particles for 1 h at room temperature shaking. The bead-phage complex was magnetized and washed five times with PBS 0.1% Tween20 and five times with PBS. Phage particles were eluted from the beads by re-suspending the washed beads in 1.5 ml of DMEM 30 mM biotin and incubating for 2 h at room temperature shaking. The beads were removed using a magnet and the supernatant used to infect a 10 ml culture of TG1 *E. coli* grown to OD_600nm_: 0.4–0.5 in 2xTY medium. Every aliquot of bacteria infected with phage was split; a portion was serially diluted and plated on 2xTY-agar/Amp/Gluc plates to assess phage titre. The remaining bacteria were spread on a Bio-Assay dish for the generation of the bacteria glycerol stock for the next round of biopanning, as described in the previous section.

### Screening of phage display scFv library

Individual colonies plated on 2xTY-agar/Amp/Gluc from the glycerol stock of the previous round of biopanning were grown in 4 ml of 2xTY/Amp/Gluc liquid culture and the plasmid purified with the Qiagen miniprep kit. Approximately 300 ng of extracted DNA from each colony was screened on agarose gel after SfiI and NotI restriction digestion.

Expression of soluble scFv from positive bacteria colonies was obtained by re-suspending 1 ml of an overnight single colony culture into 1 ml of 2xTY, 100 µg/ml ampicillin and 1 mM isopropyl β-d-1-thiogalactopyranoside (IPTG) and cultured at 30 °C for further 16 h shaking at 225 rpm.

SupT1 cells were engineered to overexpress the human transmembrane protein CD160 and after centrifugation at 3300×*g* for 10 min, 200 µl of the induced bacteria supernatant was used as primary antibody for staining 105 CD160 positive supT1 cells followed by 1 µl of anti c-myc-DyLight549 (9E10, Bio-Rad). Cells were acquired on a BD Fortessa x20II flow cytometer. Anti CD160 antibody (BY55-PE, BD Pharmigen) was used as positive control and either induced bacteria supernatant from previous round of biopanning or 2xTY medium as negative control for the staining. Positive clones were subsequently cloned in to an SFG plasmid as secreted scFv fused to mouse IgG2a Fc and transfected in HEK293T cells for transient protein production. Specific binding of the produced scFv supernatant was assessed on CD160 positive and negative supT1 cells stained with 1 μl of anti-mouse-IgG-PE (Jackson Laboratories).

### Affinity measurement

The binding kinetic of the selected scFvs were studied at 25 °C by SPR technology using a Biacore T200 machine. Supernatant from HEK293T cells transfected with different anti CD160 scFv-mIG2aFc was harvested at 48 h and immobilized on a CM5 sensor chip using a mouse antibody capture kit (GE, healthcare). The purified CD160 analyte (Sino Biological) was dialysed against the experimental running buffer HBS-EP + (GE, healthcare) prior to use. All steps were performed at a flow rate of 30 μL/min and the analyte was injected in a single-cycle kinetic mode at five different concentration (3.7 nM, 11.11 nM, 33.33 nM, 100 nM and 300 nM). The binding data were double-referenced using the interspots (representing unmodified chip surface) and the in-line buffer blank (“0 nM CD160” injection). The double-referenced sensorgrams were locally fit using the 1:1 Langmuir binding model for the extrapolation of K_a_ (on rate), K_d_ (off rate) and KD (affinity), using the BiaEvaluation software Version 3.0.

### Cytotoxicity and cytokine release assay

Peripheral blood mononuclear cells (PBMCs) were isolated from 4 healthy donors leucocyte cones (NHSBT) by Ficoll (Sigma-Aldrich) density centrifugation using SepMate PBMC Isolation tube (STEMCELL). VSV-G-pseudotyped lentivirus encoding the chimeric antigen receptors were produced in HEK293T cells by transient transfection with a 1:2:4 ratio respectively of plasmids encoding for VSV-G envelope (pMD2.G, Addgene #12259), gag-pol (gift of Elio Vanin, Baylor College of Medicine) and the transfer vector.

Before transduction, T cells were depleted of CD56-expressing NK cells using EasySep human CD56 Positive Selection Kit II (STEMCELL) according to the manufacturer’s instructions. The PBMCs were transduced 72 h after stimulation with anti-CD3/anti-CD28 antibodies (Miltenyi) and 100 U/mL human IL-2 (GeneScript) and cultured in complete media RPMI1640, 1% glutamax and 10% FCS. The cells were transduced on retronectin (Takara) coated 6-well plates at 1.2 × 10^6^ cells/well in complete RPMI1640 media, 100 U/mL human IL-2, 4 mL of lentiviral supernatant and were spun at 1000 g for 40 min.

5 × 10^4^ SupT1 wild-type (NT) or SupT1 CD160-positive cells were used as target cells and co-cultured with CAR-engineered T cells to achieve an effector:target ratio of 1:2, 1:4 and 1:8 in 200 μL of complete media. CAR-mediated cytotoxicity was assessed by flow cytometry at 24 h and 72 h timepoints. T cells were identified from target cells by staining for human CD3 (UCHT1, Biolegend) and human CD2 (RPA-2.1, Biolegend) and target cells were gated by their lack of huCD3 and huCD2 expression. Viability was assessed with live/dead cell exclusion dye eFluor780 (Thermo Scientific). Viable target cells were enumerated for each co-culture condition by acquiring a fixed amount of counting-beads (CountBright, Thermo Scientific) and the percentage cytotoxicity was calculated by normalising the number of viable target cells to that recovered from co-cultures carried out with non-transduced T cells.

Supernatants from the 72 h timepoint were harvested and used to measure the total amount of interferon-gamma (IFN-γ) and IL-2 produced. Cytokine concentrations were measured using human IFN-γ ELISA MAX kit (Biolegend) and human IL2 ELISA MAX kit (Biolegend) following the manufacturer’s protocol.

Statistical analysis was performed by two-way ANOVA with Dunnett’s post-test for comparison between CARs within the same target group. *p < 0.1, **p < 0.001 and ***p < 0.0001.

## Supplementary information

Supplementary Figures.

Supplementary Table S1.

Supplementary Table S2.

## Data Availability

The datasets generated during and/or analysed during the current study are available from the corresponding author on reasonable request.
