# Supplementary material for: A primer set for the rapid isolation of scFv fragments against cell surface antigens from immunised rats
Source: Sci Rep. 2020 Nov 5;10:19168. doi: 10.1038/s41598-020-76069-3 (PMC7644676; doi:10.1038/s41598-020-76069-3)
Supplement: Supplementary file 1 — Supplementary Figures. [file 41598_2020_76069_MOESM1_ESM.pdf]

A primer set for the rapid isolation of scFv fragments against cell surface antigens from immunised rats

Francesco Nannini[1], Farhaan Parekh[1], Patrycja Wawrzyniecka[1], Leila Mekkaoui[1], Matteo Righi[1], Fatemeh V. Dastjerdi[2], Jenny Yeung[2], Claire Roddie[1], Yuchen Bai[3], Biao Ma[3], Mathieu Ferrari[3], Shimobi Onuoha[3], Kerry Chester[2]*, Martin Pule[1,3]*

Supplementary data

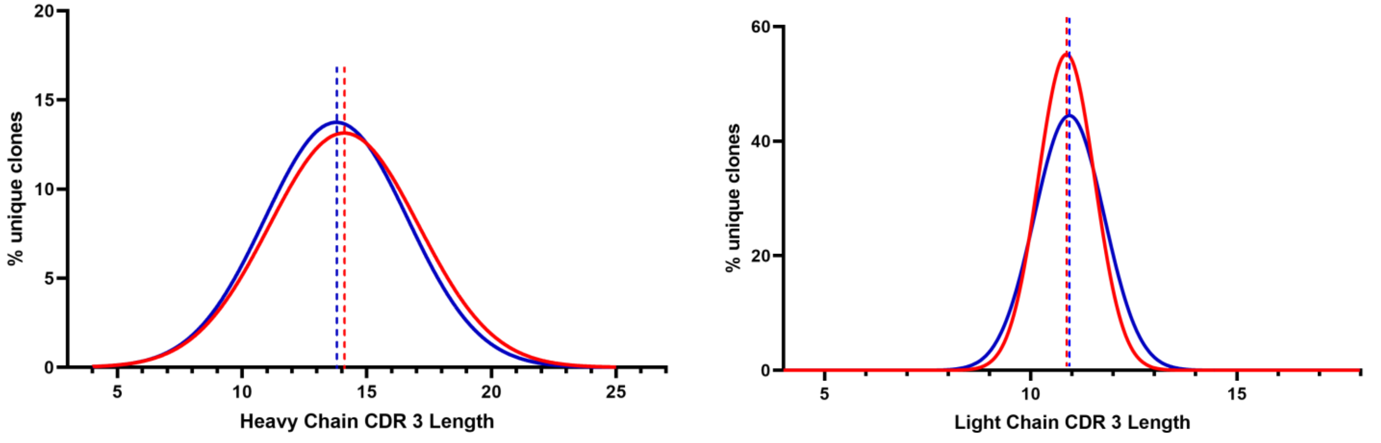

Supplementary Figure S1. CDR3 length distribution of 5'RACE and primer set amplified in naïve rats.. The length-distribution patterns of unique CDR3 sequences is represented as nonlinear-curve fitting (Gauss function) to the frequency of HCDR3 (left) and LCDR3 (right) in both the data sets. The vertical dotted lines indicate the mean of length in each group and the different colours represent the two data sets obtained from the amplification with either 5'RACE (red) or the primer set (blue).

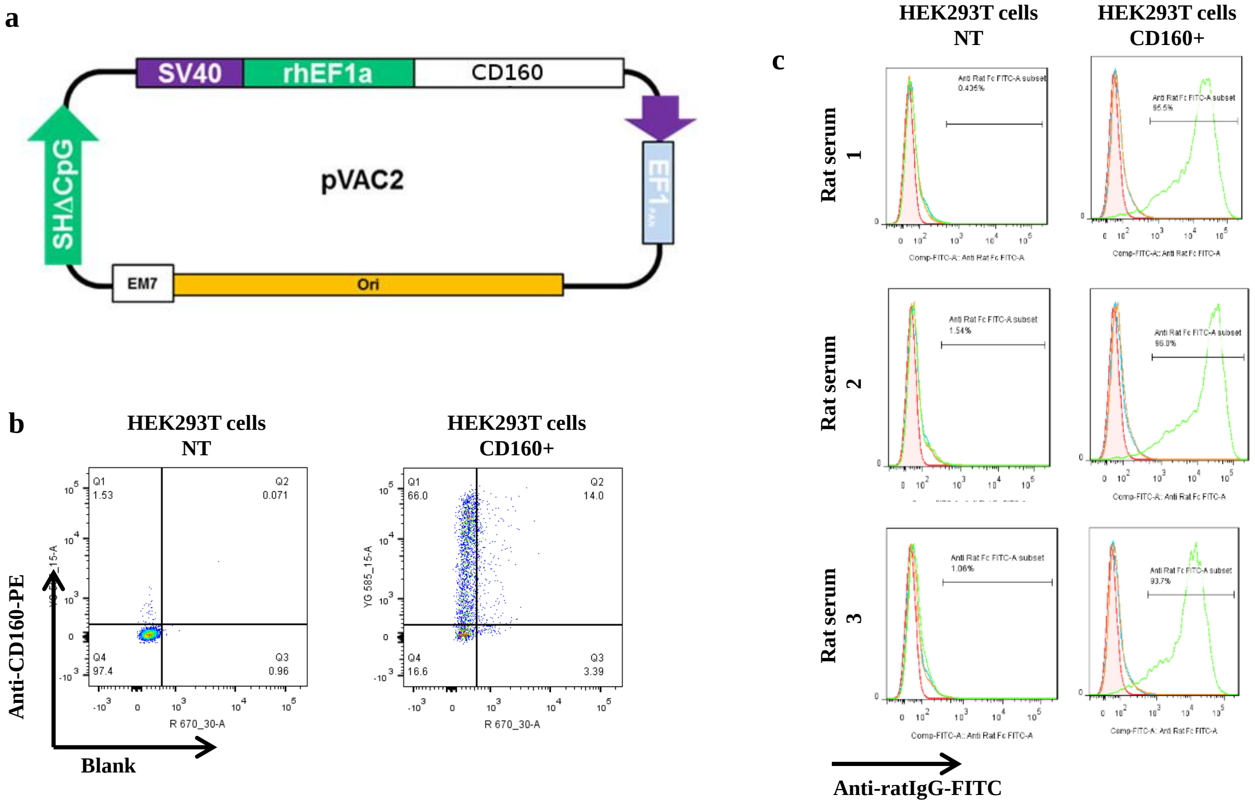

Supplementary Figure S2. Genetic vaccination with pVAC2 plasmid expressing human CD160. (**a**) pVAC2 plasmid map for the expression of codon optimized human CD160 GPI-anchor protein. The plasmid contains a CpG-free bacterial promoter (EM7) and a CpG-free Zeocin-resistance gene (Sh-ΔCpG). The EF1α gene promoter and its polyadenylation signal (EF1pAN) combined with SV40 enhancer drive expression of the transgene in mammalian cells. (**b**)pVAC2 expression in transiently transfected HEK293T cells. Cells were stained for flow cytometry with anti-CD160 antibody using non-transfected cells (NT) as a negative control. (**c**)Flow cytometry analysis of the rat serum pre- and post-vaccination against HEK293T cells NT or transfected to express human CD160. Cells were run unstained (red line) stained with anti-rat alone (blue line), pre-vaccination serum (orange line) and post vaccination serum (green line).

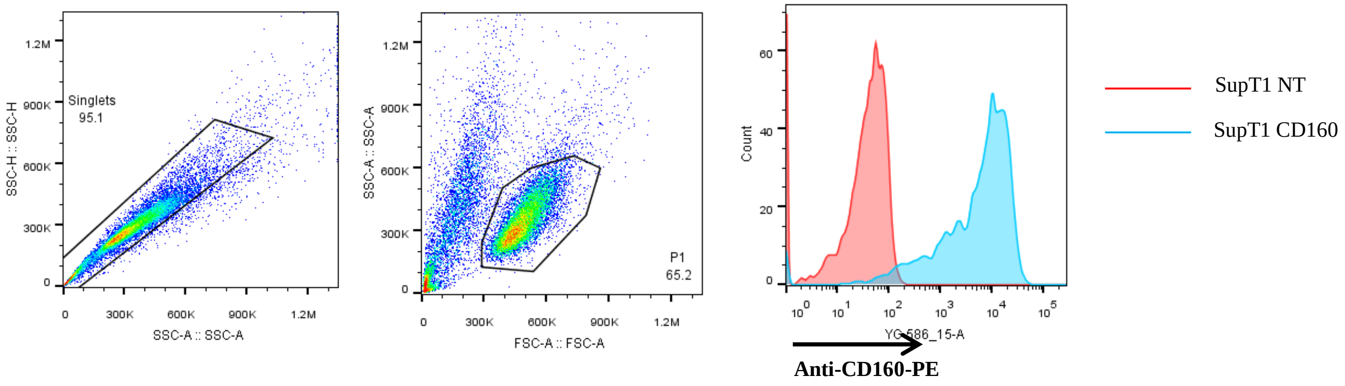

Supplementary Figure S3. SupT1 cells overexpressing human CD160. SupT1 cells were virally transduced with an SFG vector encoding human CD160-GPI to express high levels of the molecule on the cells surface. Flow cytometry histograms of SupT1 NT (red) and SupT1 SFG.huCD160 (blue) stained with commercial anti-CD160 (BY55)-PE.

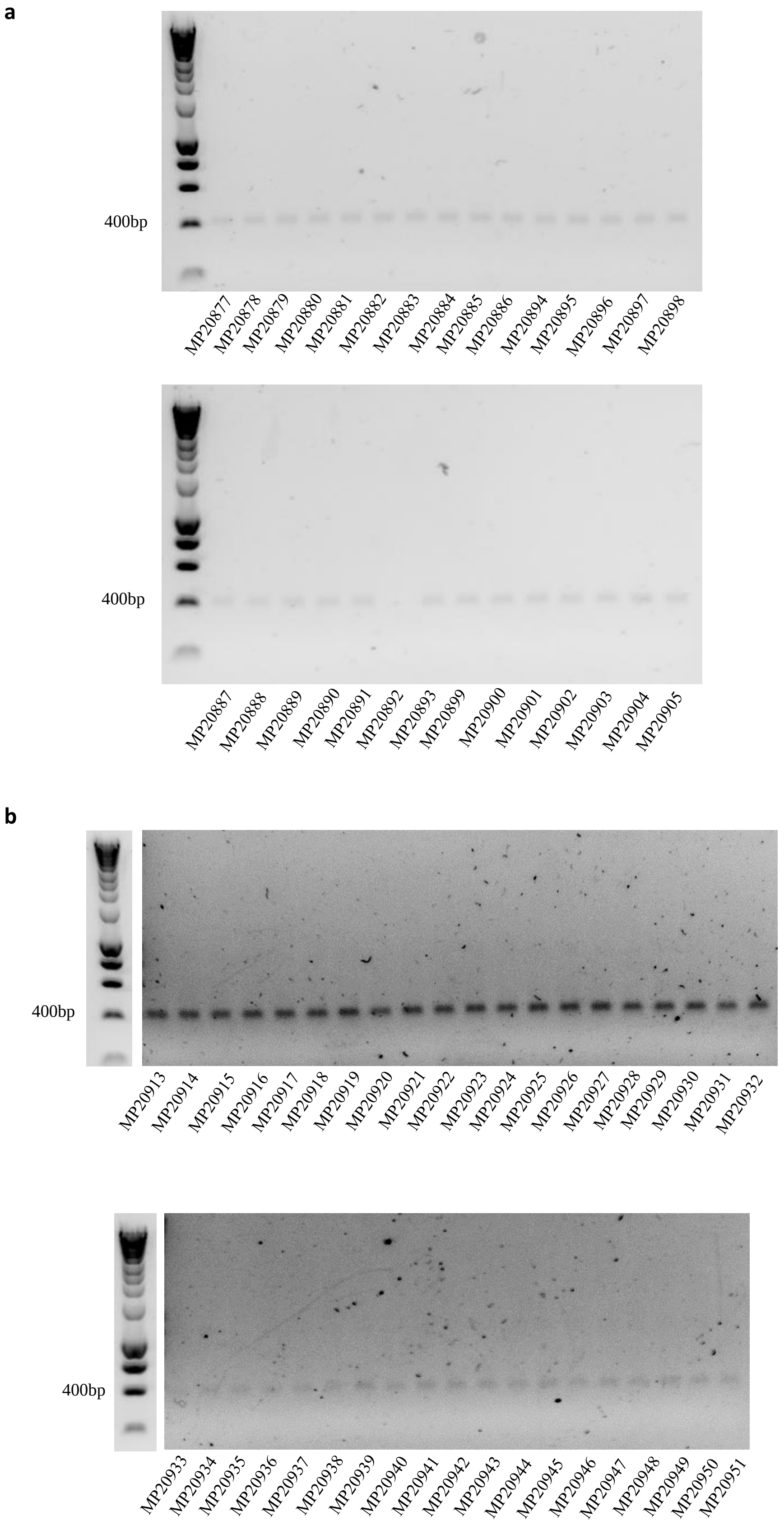

Supplementary Figure S4. Amplification of rat's VH and VLK for the assembly as scFv library. PCR amplification of the 29 primers for the VH (**a**) and the 39 primers for the VLK genes (**b**). A 400bp DNA band represented successful amplification.

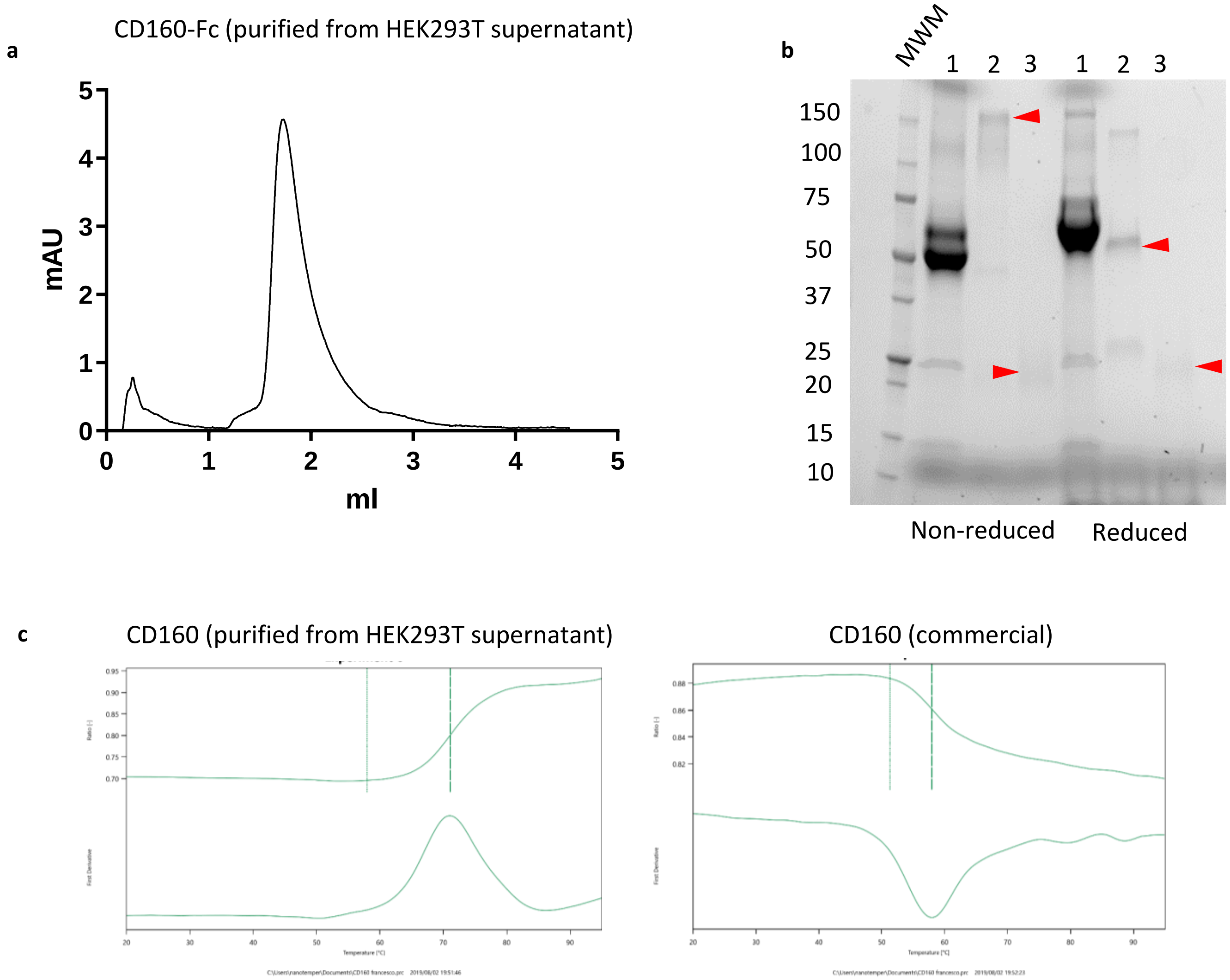

Sample ID	Onset #1	Inflection Point #1	ELISA EC50 (ug/ml)	Aggregation (%)
Commercial CD160	54.1°C	62.6°C	0.14	TBD
Purified CD160 mIgG2a Fc	55.1°C	70.8°C	0.21	3.59
HEK supernatant CD160 mIgG2a Fc	N/A	N/A	0.06	N/A

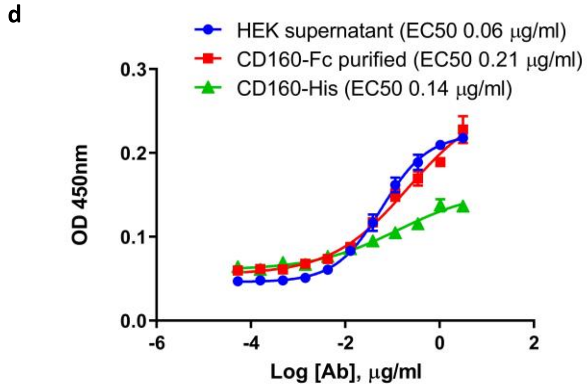

Supplementary Figure S5. Analysis of CD160 reagents used for panning. (**a**) Supernatant used for panning the library was purified via the Fc region of the protein, Size exclusion chromatography highlighted the purity of the protein and showed a homogenous peak at the expected elution volume. (**b**) SDS-page analysis of different sources of CD160 molecule. The purified protein (2) could be detected at the same molecular weight as a corresponding band within the HEK293T unpurified-supernatant (1). Commercial source of CD160-his (3) was used for comparison. (**c**) Differential scanning fluorimetry (DSF), showed that the protein obtained was stable and had an apparent melting temperature above that observed with commercially obtained protein. (**d**) Three sources of CD160 protein, commercially sourced, unpurified from supernatant and purified were used as coating antigens in ELISA experiments. Comparable EC50 profiles were obtained with all the antigen tested using BY55-huIgG1 as primary antibody for the detection.
